# Applying Antimicrobial Strategies in Wound Care Practice: A Review of the Evidence

**DOI:** 10.1111/iwj.70684

**Published:** 2025-05-26

**Authors:** Joanna Blackburn, Karen Ousey, Mark Rippon, Alan Rogers, Irena Pastar, Hadar Lev‐Tov

**Affiliations:** ^1^ Institute of Skin Integrity and Infection Prevention, School of Human and Health Sciences University of Huddersfield Huddersfield UK; ^2^ Wound Care Monash University Melbourne Australia; ^3^ Independent Wound Care Consultant North Wales UK; ^4^ Dr. Phillip Frost Department of Dermatology and Cutaneous Surgery, Wound Healing and Regenerative Medicine Research Center University of Miami, Miller School of Medicine Miami Florida USA

**Keywords:** acute and chronic wound care, antimicrobial resistance, antimicrobial stewardship, DACC technology, DACC‐coated dressings non‐medicated, wound dressings

## Abstract

Antimicrobial resistance is increasing due to an overreliance on antimicrobials to treat and manage infections. Chronic wounds are particularly vulnerable to infections and harbour complex microbial communities, increasing the risk of secondary infections caused by antimicrobial resistant bacteria. Accurate and early diagnosis of infection ensures appropriate treatment interventions and a reduction in the likelihood that antibiotic use is required. Despite this, the overuse of antibiotic treatment in wound care is still evident. Antimicrobial stewardship describes a structured approach to managing antimicrobial resistance through educating healthcare professionals about antimicrobial use to improve patient outcomes and minimise the spread of infections. However, the evidence suggests that healthcare professionals experience barriers when attempting to implement such strategies in their practice. It is essential that the principles of antimicrobial stewardship are embedded into wound care treatment and management. This review aimed to explore the current barriers to antimicrobial stewardship in wound care clinical practice and discuss the strategies that can be applied to successfully maximise infection prevention. There is a need to further educate wound care practitioners about antimicrobial stewardship and future research should concentrate on understanding how healthcare professionals can work collaboratively to implement such strategies in their practice.

1

**Summary**

- Antimicrobial resistance (AMR) is a complex problem affecting the human, animal and environmental sectors and is particularly problematic in healthcare due to the overuse and overreliance on antimicrobials.
- Antimicrobial Stewardship (AMS) can manage the impact of AMR by introducing strategies to use antimicrobials judiciously.
- Limited knowledge, education and organisational resources limits the effectiveness of AMS interventions.
- A collaborative approach to AMS is most effective and education targeting the timely and accurate diagnosis of wound infection can support the success of AMS.
- AMS is a continuous process and should focus on monitoring, surveillance and prevention.

## 2 Introduction

Antimicrobial resistance (AMR) is a complex global public health issue affecting all humanity across the human, animal, and environmental sectors. AMR happens when microorganisms undergo genetic modifications due to repeated exposure to antimicrobial substances and devices, such as antibiotics, antiseptics, antifungals, antivirals, antimalarials, and anti‐helminthics [1]. AMR disproportionately affects low‐and middle‐income countries (LMICs), often due to poor sanitation and hygiene practices, inadequate resources, and disparate access to vaccinations [2]. The World Health Organisation (WHO) [3] regards AMR as one of the top 10 global public health threats facing humanity by disrupting sustainable development goals (SDGs) which means that 28.3 million people could be forced into extreme poverty by 2050 due to increased treatment costs to treat bacteria‐associated diseases [3]. In the 79th session on Global Health and Foreign Policy, the United Nations General Assembly [4] reaffirmed the 17 SDGs and made a commitment to a 10% reduction in global deaths associated with bacterial AMR by 2030. Targets for infection prevention and control (IPC) were also set, including all countries having access to basic water supplies, sanitation, hygiene, and waste management services, in addition to the target of 90% of countries meeting WHO minimum requirements for IPC programmes by 2030. These international goals relate directly to wound care. For example, a recent meta‐analysis demonstrated that the worldwide incidence of surgical site infection (SSI) among patients was found to be 2.5% (95% CI: 1.6, 3.7) [5]. In another example, Kisibo et al. [6] estimated that the risk of infection increased following orthopaedic surgery, with 1.4%–41.9% of cases being affected by SSI. Furthermore, the prevalence of SSI following foot and ankle surgery was 4.4%, according to Cheng et al. [7].

Prevention of the spread of disease and reducing the likelihood that antibiotics will be required to treat an infection is at the core of combating AMR. The importance of prevention is highlighted by Naghavi et al. [8] who estimated that 4.71 million deaths were associated with bacterial AMR between 1990 and 2021. Whilst deaths decreased by over 50% in children < 5 years of age, they also increased by over 80% for adults 70 years and above. Naghavi et al. [8] further predicted that there could be almost two million deaths due to AMR, and over eight million deaths associated with AMR globally by 2050, if left unmanaged.

Clearly, infections, and therefore the inevitable use of antimicrobials, are common in people with wounds and there is a global call for developing strategies to manage this problem and use antimicrobials judiciously. However, there is a lack of research exploring how AMR can be minimised in wound management, or the solutions to overcome this. Therefore, this review aimed to explore the current barriers to optimising the use of antimicrobials in wound care and the strategies that can be implemented in clinical practice to maximise infection prevention.

## 3 Antimicrobial Stewardship (AMS) and its Importance in Healthcare for Managing AMR

One way of managing AMR is through Antimicrobial Stewardship (AMS). The concept of stewardship was first applied to healthcare by McGowan and Gerding [9] and is recognised as one of the three key pillars of an integrated approach to strengthening healthcare systems. Together with IPC, early intervention and IPC, and medicine and patient safety, AMS aims to educate healthcare professionals (HCPs) about AMR to ensure the optimisation of antimicrobials and work towards the goal of universal health coverage.

AMS has been defined by The National Institute of Health and Clinical Excellence [10] and WHO [11], emphasising the important role of HCPs working together across multiple specialities and disciplines to achieve a common goal (Box 1). The WHO Global Framework for the Development and Stewardship to Combat AMR [12] introduces strategies to involve multiple sectors and stakeholders in the design and implementation of AMS policies, legislation, and research. It also includes the surveillance of antibiotic‐resistant infections, promoting responsible prescribing and use of antibiotics and infection control measures to prevent the spread in both humans and animals (Box 2). The plan focuses on a *‘One Health’* approach and recognises the impact that AMR has across multiple expanses including veterinary medicine, agriculture, and the economy.

BOX 1 **NICE and WHO definitions of AMS.**

1

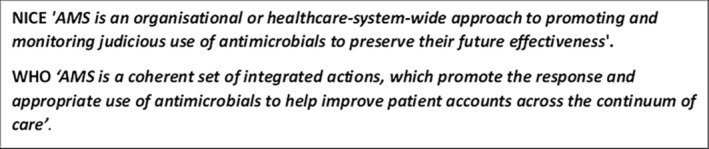

BOX 2 **One health definition.**

1 One Health is a term used to describe a principle which recognises that human and animal health are interconnected, that diseases are transmitted from humans to animals and vice versa and must therefore be tackled in both. The One Health approach also encompasses the environment, another link between humans and animals and likewise a potential source of new resistant microorganisms. This term is globally recognised, having been widely used in the EU and in the 2016 United Nations Political Declaration on AMR.

## 4 Antimicrobial Stewardship Programmes (ASPs)

AMS is supported by Antimicrobial Stewardship Programmes (ASPs); structured initiatives to promote the appropriate use of antimicrobials. The Organisation for Economic Co‐operation and Development (OECD) report ‘Stemming the Superbug Tide’ [13] stated that implementing ASPs could save up to 1.6 million lives by $2050 and $4.8 billion annually in 33 OECD countries. ASPs advocate the effective and appropriate treatment of infections to improve health outcomes and reduce the risk of AMR by encouraging clinicians to use antimicrobials judiciously and prevent unnecessary prescribing [10] and is a globally recognised strategy. In the United States of America (USA), The Centres for Disease Control and Prevention [14] introduced the Core Elements of Hospital AMS programmes guidance, requiring all hospital to implement AMS to reduce the impact of AMR. The implementation of ASPs involves a multidisciplinary approach with sustained commitment from all stakeholders involved to maintain consistency and effectiveness in treatment [15] There are several key components of ASPs:

- Optimisation of drug selection, dosage and duration, and route of administration
- Monitoring and evaluation
- Education and training

## 5 The World Health Organisation (WHO) Guidance on Implementing AMS Activities

WHO policy guidance on AMS activities [16] provides evidence‐based recommendations for implementation, promoting an integrated approach to preserve antimicrobials and seeks synergy and efficiency across essential areas of the human health sector, at all levels. The guiding principles include:

- Give due consideration to national and local context and the structure of the health system in carrying out AMS activities.
- Focus on prioritising implementation of activities that are likely to provide the greatest benefits based on national and facility needs assessment.
- Strengthen and use existing national and subnational platforms and coordinating mechanisms and resources to implement integrated AMS activities.
- Ensure strong and effective linkages and synergies between the relevant areas and disciplines related to AMR, including national infectious diseases and infection prevention programmes such as HIV, tuberculosis (TB), malaria, sexual, reproductive, maternal, newborn, child, and adolescent health, and the UHC agenda at all levels.

These principles are supported by 12 interventions represented by five pillars (Box 3). While these pillars have been widely adopted due to their systematic approach to tackling AMR, their effectiveness is dependent on leadership and local resources. For example, in low‐and middle‐income countries (LMICs), there may be insufficient infrastructure to fully implement ASPs and there may be a lack of surveillance or comprehensive data collection or reporting systems for monitoring, limiting the effectiveness in monitoring resistant trends. Education alone may not be sufficient to change behaviour in the long term, and without continuous reinforcement, clinician adherence to AMS practices may decline over time.

BOX 3 **WHO 5 pillars.**

1 **PILLAR 1: Establish and develop national coordination mechanisms for antimicrobial stewardship and develop guidelines**

1. Establish and maintain a national coordinating mechanism for AMS that is functional at national subnational and district levels.
2. Develop national treatment and stewardship guidelines, standards and implementation tools.

**PILLAR 2: Ensure access to and regulation of antimicrobials**

3 Improve access to essential, quality‐assured, safe, effective and affordable antimicrobials.
4 Regulate social triggers and remuneration policies that promote responsible antimicrobial prescription and dispensing behaviours.
5 Legislate and regulate responsible and appropriate use and disposal of antimicrobials.

**PILLAR 3: Improve awareness, education and training**

6 Improve awareness and engagement to support behavioural change of antimicrobials use.
7 Strengthen health worker capacity through the provision of tailored education and training packages according to health worker roles and functions.

**PILLAR 4: Strengthen water, sanitation and hygiene and infection prevention and control**

8 Enhance WASH in health facilities and communities.
9 Implement IPC core components in health facilities.

**PILLAR 5: Surveillance, monitoring and evaluation**

10 Surveillance of Antimicrobial Use and Consumption
11 Surveillance of AMR
12 Monitoring and Evaluation of AMS Activities

## 6 ASPs in Wound Management

ASPs are valuable in wound management to assist practitioners in understanding when antimicrobials should be used for the treatment of infectious diseases, infection prevention, and monitoring. The skin, as the largest organ of the body, can present challenges when attempting to maintain its integrity and prevent infection occurring. For example, according to Mengistu et al. [5] in their meta‐analysis, the worldwide incidence of SSI among patients was found to be 2.5% (95% CI: 1.6, 3.7). A total of 3% of the 2230 caesarean sections (CS) performed during the study by Alvi et al. suffered post‐surgical SSI [17]. The combined prevalence of SSI in patients undergoing knee surgery was found to be 3.0% according to Mahdiabadi et al. [18]. As such, it is critical that all HCPs working with patients at risk of impaired skin integrity, or those who have a wound, understand and are able to embed timely ASPs.

There is a lack of research exploring barriers to AMS in wound management or solutions to overcome this. Clinical wound infections, defined as ‘the invasion of a wound by proliferating microorganisms to a level that invokes a local and/or systemic response in the host’ [19] are a significant challenge in wound care and infection prevention is crucial to avoid delayed wound healing, reduce complications and higher healthcare costs [20]. It is important to distinguish between infection and colonisation, the latter referring to the presence of multiplying organisms in the absence of an immune response from the host [21]. Understanding colonisation is important for AMS in wound care, as whilst the process does not necessarily cause infection, it is a key indicator of the potential for infection if the colonising microorganisms become pathogenic. To minimise these risks, accurate and early detection of infection is key to ensuring successful treatment outcomes and to reduce the likelihood of antibiotic use for infection management. Chronic wounds, such as diabetic foot ulcers, venous leg ulcers, and pressure ulcers, are particularly vulnerable to infections. Biofilm‐based wound care has been proposed as a strategy to reduce bacterial load in chronic, non‐healing wounds [22]. This approach involves different methods of debridement and cleansing, followed by using modern wound dressings. Wound hygiene measures including debridement, exudate management, the use of non‐antimicrobial agents and early intervention, are essential for reducing bioburden and preventing infection, which collectively, can help reduce reliance on systemic antimicrobial medications. Surgical wounds also pose a risk of surgical site infection (SSI) or sepsis. Preventive strategies focus on maintaining proper wound and hand hygiene, using sterile dressings, and ensuring adequate nutrition to support wound healing.

Chronic wounds are characterised by complex microbial communities, and it is widely accepted that all chronic wounds are colonised regardless of aetiology and clinical signs of infection [23, 24]. Standard microbiology techniques have revealed a 50% increase in Gram‐negative AMR bacteria isolated from chronic wounds over a three‐year period [25]. Polymicrobial bioburden in wounds exists predominantly in the form of a biofilm resistant to antimicrobial treatments [26, 27, 28]. Novel technologies, including in‐depth microbiome analysis tools capable of detecting AMR genes, have also confirmed the widespread presence of multi‐drug‐resistant bacteria, including Gram‐positive methicillin‐resistant *Staphylococcus aureus* [29] and accidental pathogen *S. epidermidis* [30]. The most widely spread antibiotic resistance classes detected include resistance to beta‐lactams, aminoglycosides, and macrolide antibiotics [29]. Importantly, systemic antibiotic treatment did not affect the composition of the microbiome in the chronic wound [30], suggesting against the empiric prescription of systemic antibiotics for chronic wounds without clinical signs of infection. Resistance to widely used topical antimicrobials, including mupirocin, was also detected in 83% of samples, while multi‐drugdrug‐resistant *S. epidermidis* was associated with delayed non‐healing in patients with chronic venous leg ulcers [30]. These data confirm that chronic wounds should be considered as a reservoir of AMR bacteria, imposing a high risk of secondary infections in patients already at risk due to chronic conditions such as diabetes, immobility, advanced age, or venous insufficiency [31]. Furthermore, emerging anaerobic species challenging to cultivate and fungi were also associated with wound chronicity in diabetic patients and the risk of infection and amputation [29, 32, 33, 34]. The Food and Drug Administration (FDA) [35] has recently recognised the risk of AMR for multiple wound washes and dressings. The updated FDA guidelines categorise antimicrobials based on their level of AMR risk. High AMR concern antimicrobials, such as polymyxin B, silver sulfadiazine, and bacitracin, place products containing these ingredients in Class III. Medium‐AMR concern antimicrobials, including silver, zinc, copper, chlorhexidine, and benzalkonium chloride, place products in Class II. Finally, low‐AMR concern antimicrobials, such as parabens, hypochlorous acid, peroxide, polyhexamethylene biguanide (PHMB), and iodine, place wound care products in Class I [35].

There has been increased interest surrounding the use of a novel dialkylcarbamoyl chloride (DACC)‐coated wound dressings as a part of AMS strategies for both surgical and chronic wound care. NICE Medical technologies guidance [MTG55] [36] recommends using Leukomed Sorbact (Essity Group, Sweden), a sterile, single‐use dressing with bacteria binding properties coated with DACC, for the prevention of SSI and reducing healthcare costs associated with AMR and wound infection. The DACC‐coated wound dressing works by physically binding and removing bacteria from the wound rather than using antimicrobial agents to kill bacteria. There is a growing amount of evidence for the efficacy of DACC technology, reducing the need for antibiotic treatment and supporting AMS [37, 38, 39]. For example, Magro [40] found that using DACC‐coated wound dressings after caesarean section (CS) reduced costs, improved patient experiences, and reduced the incidence of SSI by 38%. In a narrative review of the use of DACC‐coated wound dressings, Chadwick and Ousey [41] found evidence for their effectiveness in the treatment of acute and chronic wounds. Stanirowski et al. [38] found that in a randomised controlled trial (RCT) comparing DACC‐coated surgical dressings against a standard surgical dressing following CS, the SSI rate 14 days post‐surgery was 1.8% for the intervention group and 5.2% for the standard care group, respectively (*p* = 0.04). Similar findings were reported in the study by Stanirowski et al. [39] with an SSI rate 14 days post‐surgery of 2.8% for DACC‐coated wound dressings compared with 9.8% for standard dressings, although this finding was not statistically significant. A reduction in infection rates is reflected in NICE [36] estimating an annual reduction of length of hospital stay when using DACC‐coated wound dressings of 30 bed days per 100 000 population for CS and 14 bed days per 100 000 population for vascular surgery.

In addition to being effective for wound infection, DACC‐coated wound dressings have a significant cost saving benefit; NICE [36] estimates an annual saving of £10 700 per 100 000 population when using the dressings compared to standard care. Furthermore, based on an increased length of hospital stay due to wound infection of 4 and 10 days for CS and vascular surgery, respectively, NICE [36] also estimates savings to the NHS of £5.3 million per year for CS and up to £1.2 million per year for vascular surgery when using these dressings in place of standard care. Economic analysis of data using DACC‐coated wound dressings has demonstrated the cost saving benefit of using the dressings over standard care. In the study by Stanirowski et al. [38] total costs for preventing and treating SSI were 5775 euros in the standard dressings group compared with 1065 euros in the DACC‐coated wound dressings group. Cost modelling using this data from the perspective of the NHS (National Health Service) in the United Kingdom (UK) has also shown a cost saving of £119.07 per patient when using DACC dressings [39]. Magro [40] also found that DACC‐coated wound dressings after CS resulted in significant reductions in SSI rates (38%) and readmission rates for SSI (31%), in a UK‐based study in an NHS maternity unit. The evaluation also found a 38% reduction in hospital readmissions and a 30% reduction in antibiotic use for infection management.

## 7 Challenges to the Implementation of ASPs

National policy and local guidance can provide a generalised organisational structured approach to AMS activities, but several barriers are still evident at the individual level regarding how clinicians can adhere to AMS policies and procedures. Many of these barriers are attributable to a lack of awareness or training in AMS principles, leading to the overuse or inappropriate use of antimicrobials [42, 43]. Paden et al. [44] in their qualitative study, investigated barriers and facilitators faced by specialist nurses when educating individuals with wounds in their daily practice and highlighted the importance of involving patients and relatives in wound care education to promote motivation and adherence to educational objectives. The authors also suggested using strategies such as phone apps, photography, and motivational interviewing to promote compliance. Organisational context and the availability of resources, such as insufficient medical facilities, hinder the effectiveness of ASPs [45, 46]. At an individual level, evidence suggests that clinicians are concerned and value the importance of ASPs but are also unaware of how they can be fully engaged in activities to support change. Educating nurses and empowering them to participate in decision making can significantly enhance AMS practices [47] Davey and Aveyard [48] support these findings by stating that as the nurses in their study were reluctant to verbalise their views on ASPs, their contribution towards their success was limited. Broom et al. [49] demonstrated that empowering nurses to question and challenge prescribing practices can lead to better AMS outcomes. This is reiterated internationally, with Alghamdi et al. [50] finding that inappropriate antimicrobial prescribing was attributable to ineffective local policies and procedures due to a fear of liability and limited education on AMR and AMS in three Saudi hospitals. However, Paden et al. [44] highlighted barriers to staff being actively involved in ASPs, including low patient to nurse ratios and high workloads.

Nurses are integral to the success of ASPs as they are often responsible for assessing patients for signs and symptoms of infection, monitoring responses to antibiotic treatments, and identifying potential adverse effects [51]. Ensuring adherence to prescribed antibiotic regimens and reducing the risk of resistance development [52] is a critical nursing responsibility and promotes optimal antibiotic use [53] Implementing and adhering to infection control protocols, such as hand hygiene and isolation precautions, is another key area where nurses and wound care practitioners contribute to AMS for the prevention of AMR infections [54] However, evidence also suggests that nurses feel they do not have a definitive role in AMS [55], often due to their limited capacity and capability to prescribe [56].

## 8 The Role of the Multidisciplinary Team in ASPs

The effectiveness of ASPs is largely dependent on the collaborative efforts of a multidisciplinary team including HCPs, support staff, patients, and their families and carers. Data collection methods, feedback, education, and long‐term assessment and follow‐up are some of the key factors supporting successful ASPs [57]. As discussed by Ousey and Blackburn [58], education is central to AMS and HCPs have a responsibility to be aware of AMR, undertake AMS, and be aware of the differing values of their role in ASPs. The evidence reinforces the importance of a multidisciplinary team; for example, Roger, Coulter, and Thompson [59] found that introducing an antimicrobial pharmacy technician to the ward team improves AMS, patient care, and safety while reducing the risk of AMR. Tissue viability leads, podiatrists, and infectious disease physicians can provide expertise in diagnosing and treating infections, develop and update AMS protocols and guidelines, guiding appropriate antimicrobial selection based on clinical evidence, review complex cases, and provide consultation for difficult‐to‐treat infections. Pharmacists can provide support in antimicrobial therapy and ensure safe medication use, reviewing antimicrobial orders for appropriateness, dosing, and potential interactions, and educate healthcare staff and patients on proper antimicrobial use [60]. Microbiologists provide data on local antimicrobial resistance patterns, conduct and interpret laboratory tests to identify pathogens and their susceptibilities, assist in developing targeted therapy based on microbiological data, and track and report resistance trends to inform ASPs [61]. Hospital administrators support ASPs by allocating resources, facilitating training and education, and ensuring that ASPs are integrated into hospital policies [62].

## 9 ASPs Guidelines for Implementation

Several evidence‐based guidelines have been developed to guide HCPs on how to appropriately manage and monitor AMR and ASPs, including the Start Smart Then Focus (SSTF) toolkit for ASPs in secondary care and the Treat Antibiotics Responsibly, Guidance, Education, Tools (TARGET) toolkit for ASPs in primary care.

SSTF is an evidence‐based guidance for secondary care clinicians and leaders, designed to reduce the risk of AMR while safeguarding the quality of care for patients with infection. The scope is limited to inpatient care settings (including acute, community and mental health trusts) as an environment with relatively high intensity of antimicrobial use where patients are monitored over time, facilitating review and revision of the initial diagnosis and treatment regimen. The toolkit is structured into three main parts:

- SSTF Principles and Best Practices
- Compliance and Audits
- Antimicrobial Stewardship Programmes providing detailed guidance on the implementation and management of AMS programs within healthcare facilities. It includes resources for developing and sustaining effective stewardship programmes, as well as tools for evaluating their impact and effectiveness.

The TARGET antibiotic toolkit is an educational resource to support AMS and educate HCPs, improve prescribing practices, and promote the responsible use of antimicrobials. TARGET includes evidence‐based guidelines and protocols for prescribing and specific recommendations for diagnosis, treatment, and follow‐up and supports public health campaigns that raise awareness about AMR and the importance of responsible antimicrobial use [63, 64]. The toolkit includes decision support tools such as diagnostic aids, checklists, and patient management algorithms and incorporates audit and feedback mechanisms that enable healthcare providers to review prescribing practices, supporting the identification of areas for improvement and reinforcing adherence to guidelines [65]. HCPs and patients are educated through online training modules, patient information leaflets, and videos. Research by Jones et al. [66] assessing HCP views of the toolkit found that it is considered a useful method of promoting AMS, particularly around the ability to compare their own antibiotic prescribing practices with others and the TARGET Treating Your Infection leaflet. However, time pressures, cost, accessibility, and competing priorities meant many GP staff were unable to utilise all the available resources, particularly audit and educational materials. TARGET has been used successfully in community pharmacies in England, providing patients with education on appropriate antibiotic use and positively impacting influenza vaccination uptake [67].

## 10 Wound Infection and Inflammation

**FIGURE 1:**
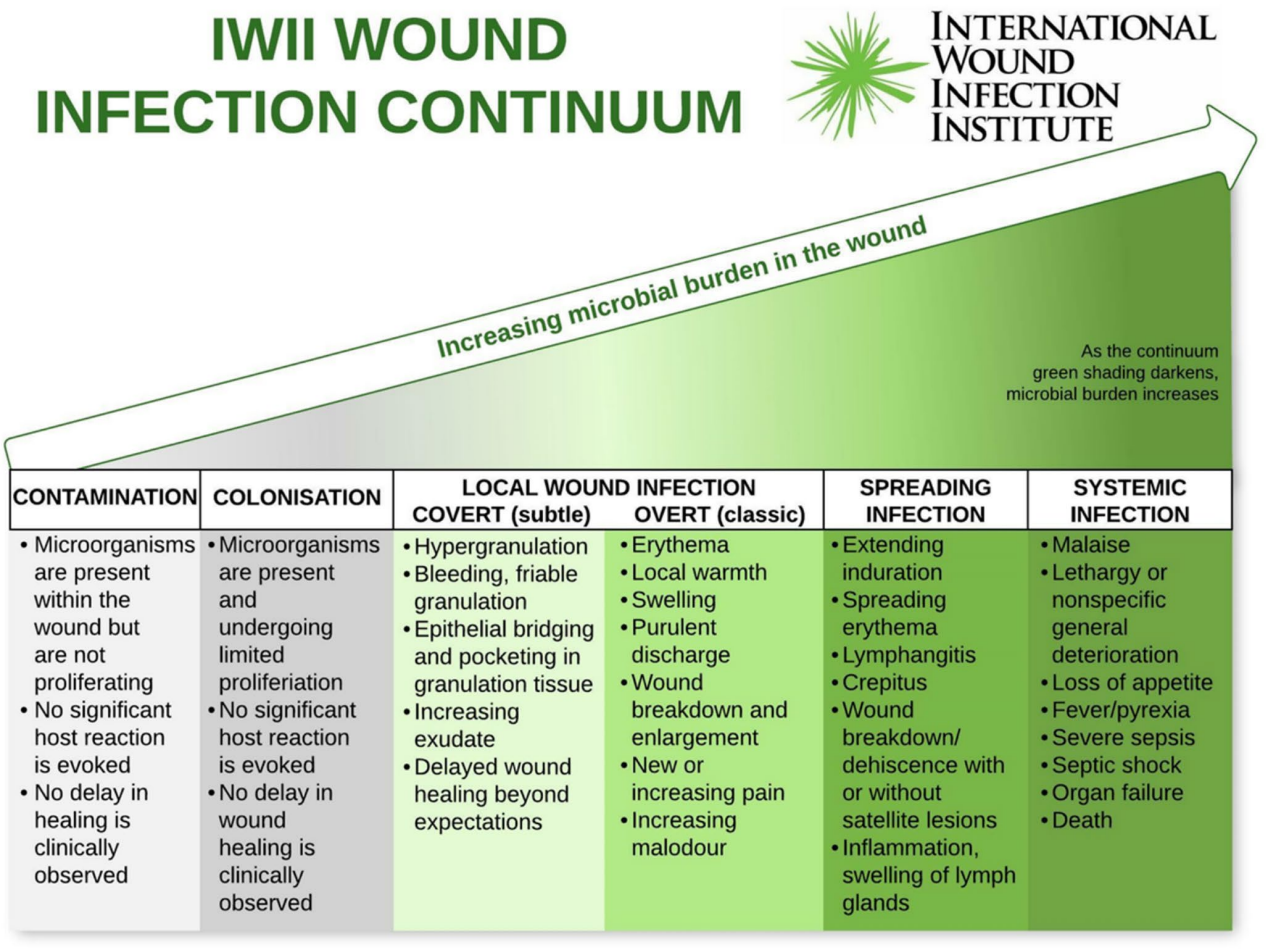
IWII WIC (Reproduced with kind permission from the IWII).

Recognising the difference between wound infection and inflammation is crucial in preventing AMR. The IWII Wound Infection Continuum (WIC) [19] exemplifies and describes the symptoms of the five phases of wound infection from contamination to systemic infection (Figure 1). Prevention is the first mechanism for controlling AMR. Appropriate sanitation and hygiene facilities could prevent a large proportion of AMR‐related deaths worldwide [2]. Ho et al. [68], in their review, emphasise the urgent need for ASPs with local guidelines that focus on the proper use of antibiotics. Inappropriate prescribing can be defined by several key factors including inappropriate indication, choice or selection of antibiotic, quality, dosage, or adherence to treatment, and a multidisciplinary approach is necessary to reduce antibiotic misuse.

Education that targets the accurate and timely diagnosis of infection should be at the centre of wound management and skin integrity [19, 69] and should be available to all healthcare staff to support a culture of stewardship and accountability [70], reducing the likelihood of unnecessary prescribing.

Quality improvement programmes can be implemented for wound assessment and management and could include ongoing reviews of outpatient and inpatient use of antimicrobials, accurate recording of documentation and measurement of clinical outcomes [58]. Comparisons against previous performances and other sites to share good practice and organisational learning [71] are also recommended. When infections are suspected on clinical grounds, rapid diagnostic tests, wound swabs, and wound cultures can be used to accurately identify causative organisms and their antibiotic susceptibility profile. There is also evidence supporting therapeutic approaches for detecting chronic wound biofilms including debridement and imaging tools [72]. When a wound infection is diagnosed, narrow‐spectrum antibiotics should be used to target specific pathogens and should be used for a limited period; repeat prescriptions should be avoided; rather, patients should be reviewed by HCPs to assess the wound for any signs of improvement or deterioration. Patients should be educated to complete the course of antibiotics and not to share antibiotics with others. Antimicrobial wound dressings should only be used for wounds that are clinically infected, and there may be some occasions when an antimicrobial is used to prevent infection in a vulnerable patient, or when there are a range of co‐morbidities present (e.g., cancer, diabetes) where wound infection needs to be prevented. DACC‐coated wound dressings can be used for prolonged periods of time as there is no risk of resistance developing. This also applies in the absence of infection signs, to control or reduce the bacterial load, which may lead to a reduced need for antibiotics [73]. In wound types that have a high risk of infection, DACC‐coated wound dressings can be used as a preventative measure, especially when signs of infection may be subtle. The Global Alliance for Infections in Surgery [74] suggests implementing a bundle for the prevention of surgical site infection (SSI) including preoperative bathing and showering, surgical antibiotic prophylaxis, hair removal, surgical hand preparation, and skin antiseptic preparation. Targeted antimicrobial therapy should be used when culture results are received and clear clinical signs of wound infections and pathogens have been identified. This is crucial in the management of specific wounds including diabetic foot ulcer (DFU) where there is a risk of osteomyelitis. In the absence of an infection diagnosis, offloading should be the preferred treatment option to reduce the likelihood of infection progression, encourage wound healing, and reduce the need for antibiotics.

**FIGURE 2:**
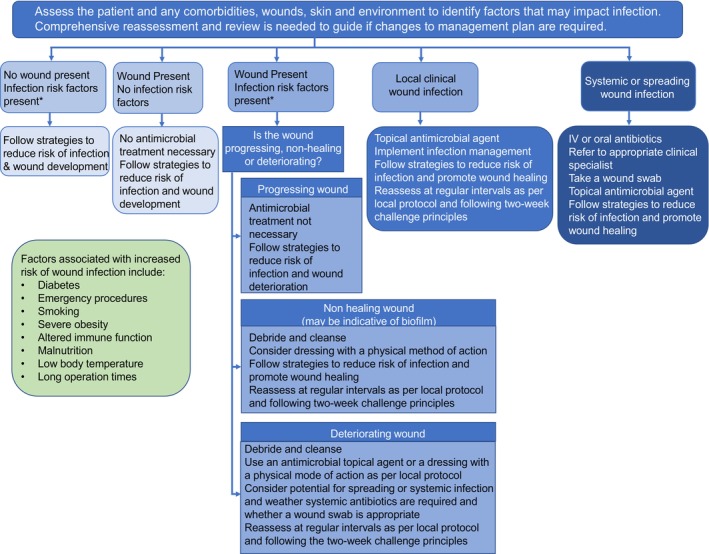
Pathway to guide the management of patients with wounds with or without infection considering the principles of antimicrobial stewardship. IV, intravenous. Figure reproduced with permission from Wounds UK Best Practice Statement: Antimicrobial stewardship strategies for wound management. Wounds UK, London.

The continuum highlights factors such as chronic diseases, wound duration and severity, and environmental factors including an unhygienic environment, moisture management, and interface pressure, which contribute to wound infection severity. Behaviour change is essential in optimising the use of antimicrobials, with Pada et al. [75] and WHO [12] emphasising the importance of hand washing and hand hygiene to prevent the spread of drug‐resistant pathogens. Timely management of infection is important for the prevention of Sepsis. Sepsis is a life‐threatening potential consequence of severe infection, accounting for approximately 48 000 deaths per year in the UK [76] reinforcing the importance of proper wound infection management. Antibiotics are central to sepsis management, and the updated NICE guidelines [77] *Suspected sepsis: recognition, diagnosis and early management*outlines the recommendations on recognition, treatment, and controlling the source of infection, monitoring, and education. Wounds UK [78] presented a pathway to assist clinicians in the management of patients with wounds, with or without infection risk (Figure 2).

## 11 Summary

**FIGURE 3:**
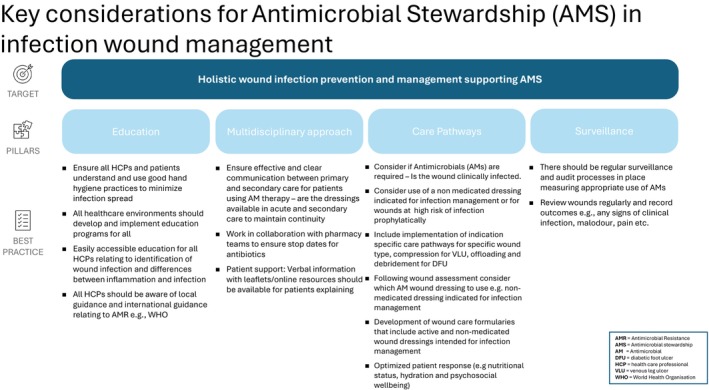
Continuous process for implementing ASPs.

AMS is a constant and evolving process with measurable outcomes including surveillance and audit procedures, and it is the responsibility of all those involved in healthcare to ensure that they are aware of local and national policies and guidelines. A collaborative approach to AMS is the most effective method of ensuring success, and wound care practitioners, HCPs, support staff, patients, and their families and carers all have an important role to play in limiting the effects of AMR. Prevention of wound infection is paramount, and there should be a focus on using products that reduce infection risk without triggering AMR. There are wounds that will become infected and require antimicrobial use; however these should be reviewed on a regular basis and only used when a holistic wound assessment demands their use. The evidence suggests there is a need to further educate wound care practitioners regarding AMS and how to implement ASPs in wound management. Future research should focus on working collaboratively with HCPs to understand how best to implement ASPs in wound care. Figure 3 describes the continuous process for the implementation of ASPs, emphasising an iterative and adaptive approach to optimising antimicrobial use. Focusing on monitoring, surveillance, and prevention, this approach highlights the importance of communication between HCPs and patients to ensure that AMS interventions remain dynamic and responsive to emerging challenges, to support improved patient outcomes, optimise appropriate prescribing, and reduce AMR.

## Conflicts of Interest

This paper was supported by a non‐restrictive educational grant from Essity.

## Data Availability

No new data were generated during the course of this research.
